# Screening of osteoprotegerin-related feature genes in osteoporosis and functional analysis with DNA microarray

**DOI:** 10.1186/2047-783X-18-15

**Published:** 2013-06-03

**Authors:** Xiaoming Wu, Shuzhang Guo, Guanghao Shen, Xing Ma, Chi Tang, Kangning Xie, Juan Liu, Wei Guo, Yili Yan, Erping Luo

**Affiliations:** 1 School of Biomedical Engineering, Fourth Military Medical University, No. 17, Changle West Road, Xi’an, Shanxi 710032, China; 2 Department of Orthopedic surgery, Affiliated Hospital of Xi'an Medical College, Xi’an 710077, China; 3 Department of Orthopaedics, First Affiliated Hospital of Medical School, Xi’an Jiaotong University, Xi’an 710061, China

**Keywords:** Osteoprotegerin, Osteoporosis, Interaction network, Module analysis, Function enrichment analysis

## Abstract

**Background:**

Osteoporosis affects 200 million people worldwide and places an enormous economic burden on society. We aim to identify the feature genes that are related to osteoprotegerin in osteoporosis and to perform function analysis with DNA microarray from human bone marrow.

**Methods:**

We downloaded the gene expression profile GSE35957 from Gene Expression Omnibus database including nine gene chips from bone marrow mesenchymal stem cells of five osteoporotic and four non-osteoporotic subjects. The differentially expressed genes between normal and disease samples were identified by LIMMA package in R language. The interactions among the osteoprotegerin gene (*OPG*) and differentially expressed genes were searched and visualized by Cytoscape. MCODE and Bingo were used to perform module analysis. Finally, GENECODIS was used to obtain enriched pathways of genes in an interaction network.

**Results:**

A total of 656 genes were identified as differentially expressed genes between osteoporotic and non-osteoporotic samples. IL17RC, COL1A1, and ESR1 were identified to interact with OPG directly from the protein-protein interaction network. A module containing ERS1 was screened out, and this module was most significantly enriched in organ development. Pathway enrichment analysis suggested genes in the interaction network were related to focal adhesion.

**Conclusions:**

The expression pattern of *IL17RC*, *COL1A1*, and *ESR1* can be useful in osteoporosis detection, which may help in identifying those populations at high risk for osteoporosis, and in directing treatment of osteoporosis.

## Background

Osteoporosis is a chronic disease involving multiple factors, and the incidence of osteoporosis in senile people and postmenopausal women is rising as the population ages, thereby adding to societal problems of health [1]. Osteoporosis affects 200 million people worldwide. Among those affected, approximately 80% are women aged 60 years or older [2]. Current treatment of osteoporosis is mainly by drugs, but it is high cost, time consuming, the drugs have many side effects, and the curative effect is not ideal. In recent years, more and more scientists have committed to stimulate stem cells to differentiate into osteoblasts for the treatment of osteoporosis.

Mesenchymal stem cells have the potential to differentiate into the osteoblasts [3] or repair bone tissue by the lineage or chondrocyte differentiation method [4]. Osteoprotegerin (OPG), a member of the tumor necrosis factor (TNF) receptor family, suppresses the coupled process of skeletal turnover. OPG functions as a decoy receptor for osteoclast differentiation factor or as a receptor activator of nuclear factor κB (RANK) ligand [5]. Osteoclast differentiation factor promotes bone resorption by enhancing the formation and activation of osteoclasts when it binds to RANK on hematopoietic osteoclast progenitor cells as well as on mature osteoclasts [6].

At present, high-throughput screening of differentially expressed genes and function identification can get the expression profile of bone marrow mesenchymal stem cells, and find its differentiation mechanism. But the study of high-throughput screening is rare due to expensive equipment and annotation probe. In this paper, based on a group of bone marrow mesenchymal stem cells in gene expression profile data, we studied marker genes closely linked with osteoprotegerin of osteoporosis in hopes of being able to treat osteoporosis through osteoprotegerin in bone marrow mesenchymal stem cells.

## Methods

### Affymetrix microarray

GSE35957 was downloaded from Gene Expression Omnibus (GEO) database (http://www.ncbi.nlm.nih.gov/geo/), which is based on GPL570 [HG-U133_Plus_2] Affymetrix Human information Genome U133 Plus 2.0 Array Platform (Affymetrix, Santa Clara, CA, USA). Microarray probe annotation information was downloaded from the Affymetrix Company, including all AffymetrixATH1(25K) gene chip probe information, and the probe annotation information files of the platform. A total of nine gene chips from mesenchymal cell samples, including five gene chips from osteoporosis patients and four gene chips from non-osteoporosis samples, were used for analysis.

### Data preprocessing and analysis of differentially expressed genes

The original data were preprocessed by Affymetrix [7,8] package in R language. LIMMA [9] package in R language was used to identify the differentially expressed genes between the expression profile of five osteoporosis patients and four non-osteoporosis samples. Multiple testing correction was performed by Bayesian method [10]. An FDR <0.01 and |logFC| >1 were chosen as thresholds for screening the differentially expressed genes.

### Prediction of interaction between differentially expressed genes

Differentially expressed genes play a role through interacting with each other. Therefore, we used HitPredict software (http://hintdb.hgc.jp/htp/) to search the differentially expressed genes that can interact with OPG gene. HitPredict is a resource for high confidence protein-protein interactions. It collects protein-protein interactions from IntAct, BIOGRID and HPRD databases; annotates these interactions; and assigns a reliability score for each interaction according to the likelihood ratio using naïve Bayesian networks combining sequence, structure and function annotations of the interacting proteins [11]. So far, HitPredict has 239584 protein-protein interactions across nine species, 168458 of which are predicted to be of high confidence. This study used the protein-protein interactions with high confidence to find interactions between the differentially expressed genes, and used the Cytoscape [12] to visualize the interaction relationships.

### Module analysis of interaction network

MCODE (Molecular Complex Detection) detects densely connected regions in large protein-protein interaction networks that may represent molecular complexes. In this study, we used MCODE to mine the modules from the protein-protein interaction network with degree >2. Further, we used Bingo [13] to annotate each module based on the hypergeometric distribution (FDR <0.05).

### Pathway enrichment analysis of interaction network

GENECODIS was used to perform biological pathway enrichment analysis of all genes in the interaction network with FDR <0.05. GENECODIS is a function analysis tool of gene, and it integrates different information resources (GO, KEGG or SwissProt), searches and arranges gene set annotation by statistical significance [14].

## Results

### Screening differentially expressed genes

**Figure 1:**
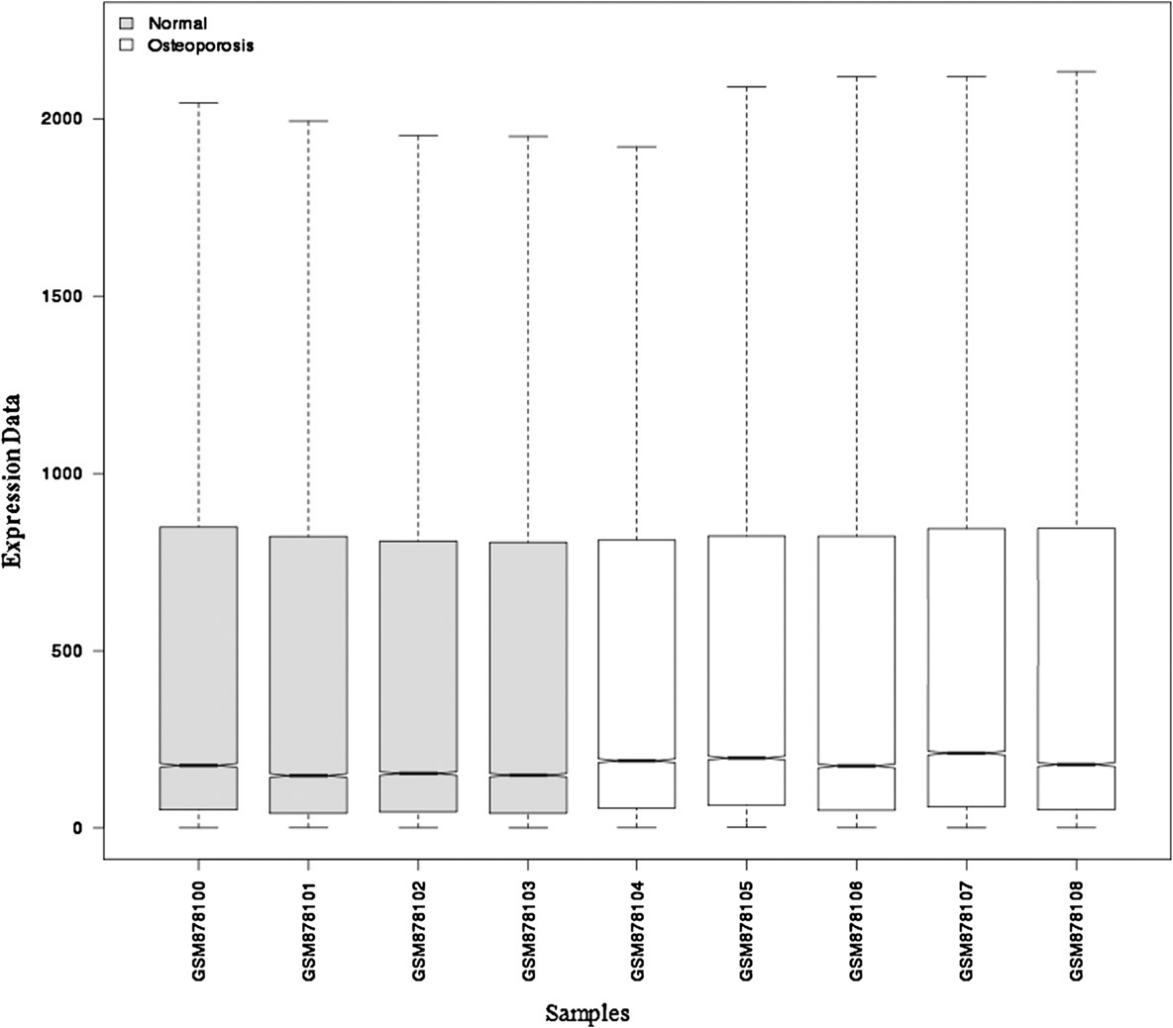
**Expression data after the standardization.** The gray box represents the four normal human bone marrow mesenchymal stem cell samples, and the white box represents five osteoporosis samples. The black line in each box is the median of data, and its distribution can determine the standardization degree of the data. When the black lines are on approximately the same level, this indicates an excellent degree of standardization.

The original data were preprocessed by Affymetrix package in R language to remove systematic bias. The results show a black line in each box at approximately the same level, which indicates an excellent degree of standardization (Figure 1). After preprocessing, the normalized expression profile data were differentially compared, and 656 differentially expressed genes exceeding the difference threshold (FDR<0.01and |logFC|>1) were screened out, including 71 downregulated genes and 585 upregulated genes.

### Prediction of interaction between OPG and differentially expressed genes

**Figure 2:**
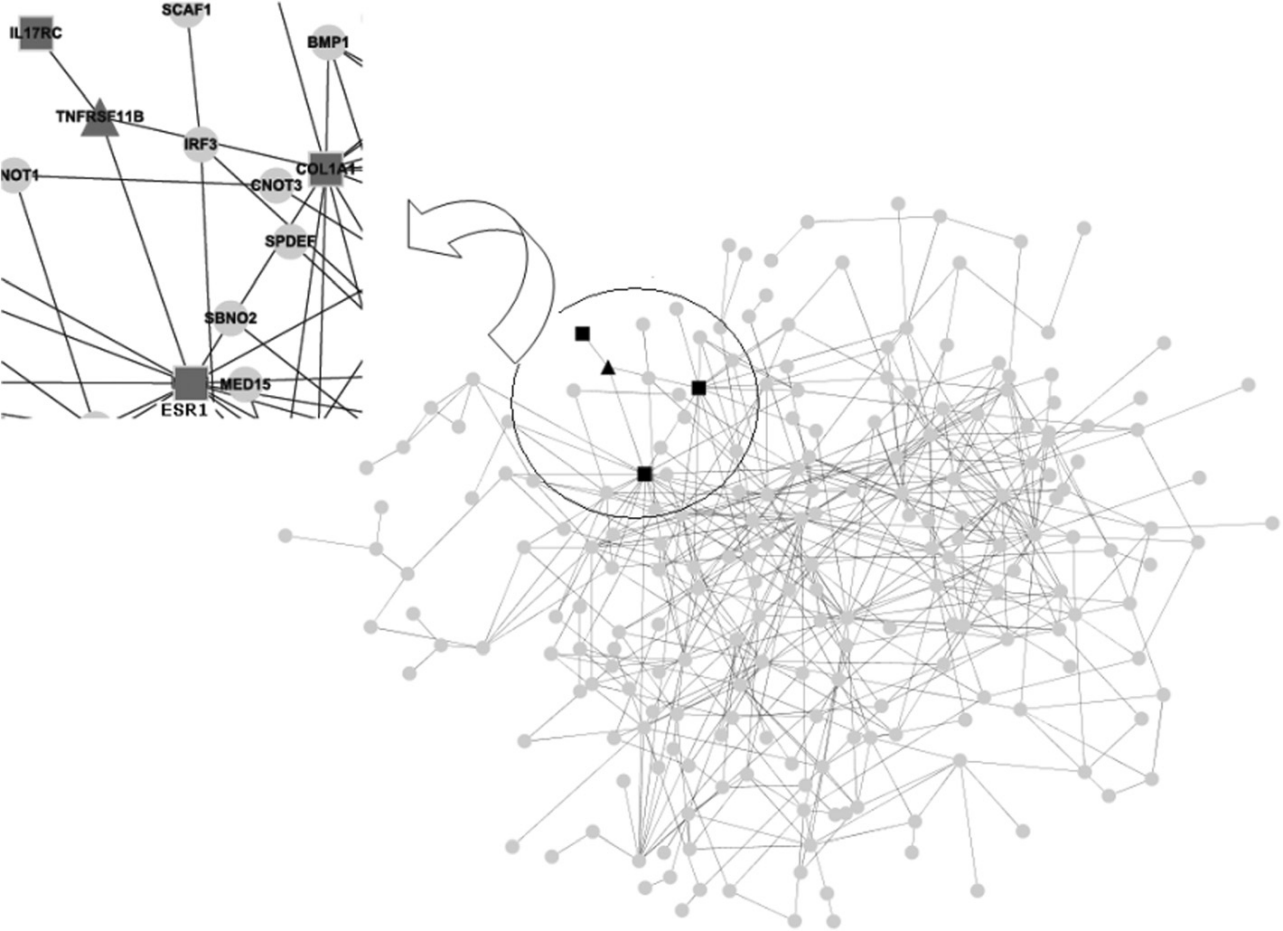
**Interaction network among differentially expressed genes and the osteoprotegerin gene (*OPG*).** The gray circular node represents the differentially expressed gene, the black triangular node represents the OPG gene and the black square node represents the gene directly interacting with OPG.

The software HitPredict was used to search all the differentially expressed genes that interacted with the OPG gene. There were 485 interactions among the 656 differentially expressed genes. Three differentially expressed genes (*IL17RC*, *COL1A1*, and *ESR1*) were found to interact with the *OPG* gene directly (TNFRSF11B) (Figure 2).

### Module analysis of interaction network

**Figure 3:**
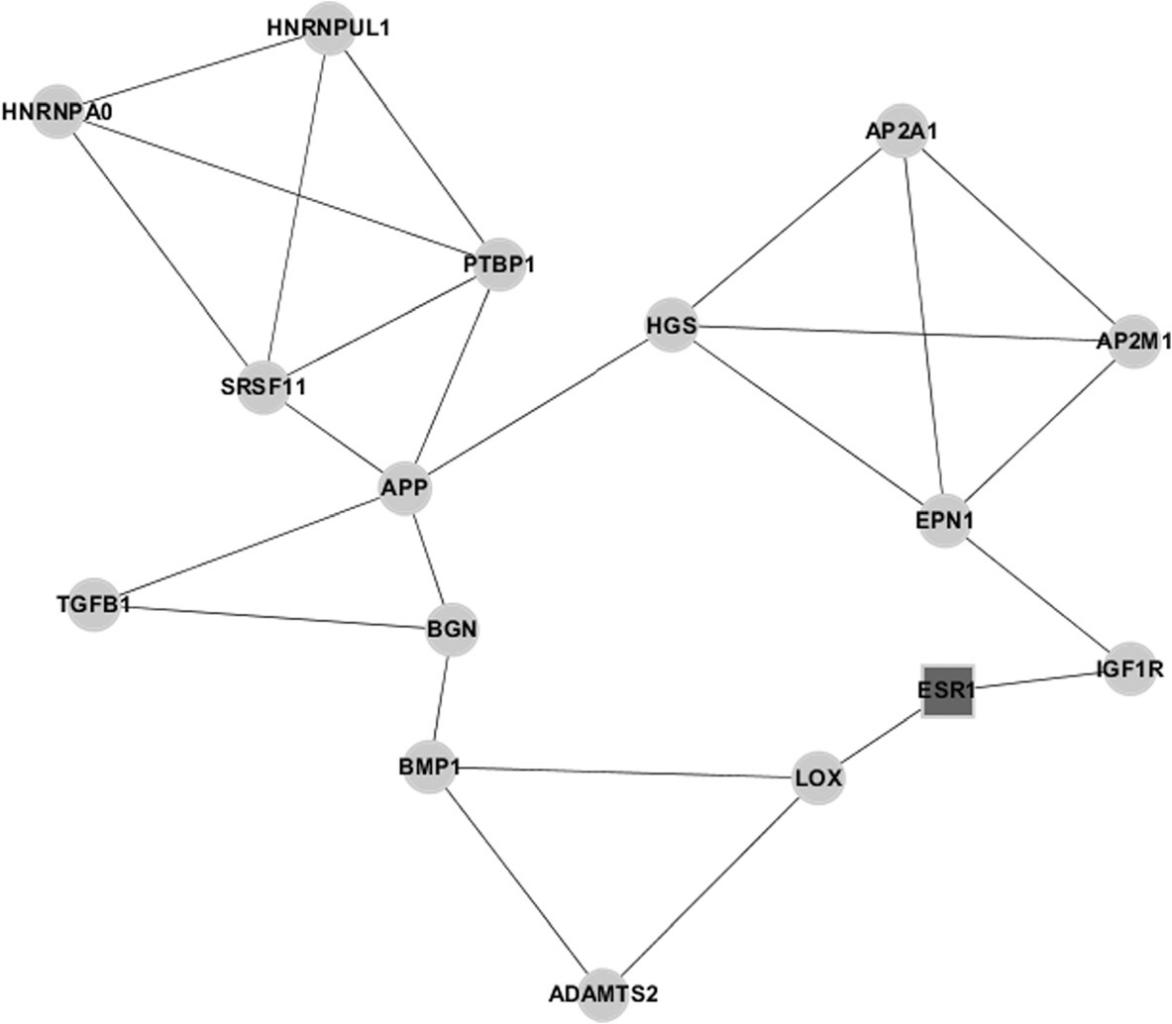
**Module diagram of *ESR1 *gene.** The square box represents the *ESR1* gene (directly interacting with the osteoprotegerin gene (*OPG*)), and the other circular nodes represent differentially expressed genes.

MCODE was used to mine the densely connected modules in protein-protein interaction network. With degree >2, one module was screened out (Figure 3). This module contains 16 nodes, and one of them is *ESR1*. By using BINGO to perform functional annotation, 16 significant GO terms were screened out, of which GO48513 (involved in organ development) is the most significant (Table 1). *IGF1R*, *APP*, *BGN*, *BMP1*, *ESR1*, *LOX*, *ADAMTS2*, and *TGFB1* genes were enriched in GO48513.

**Table 1:** List of module function

**GO-ID**	**FDR**	**x**^**a**^	**Description**	**Genes in test set**
48513	1.24E-02	8	Organ development	*IGF1R, APP, BGN, BMP1, ESR1, LOX,ADAMTS2, TGFB1*
10467	1.24E-02	7	Gene expression	*APP, HNRNPUL1, SRSF11, PTBP1, ESR1,ADAMTS2, HNRNPA*
9888	1.24E-02	6	Tissue development	*IGF1R, APP, BMP1, ESR1, ADAMTS2, TGFB1*
16070	1.34E-02	6	RNA metabolic process	*APP, HNRNPUL1, SRSF11, PTBP1, ESR1, HNRNPA*
9987	2.09E-02	16	Cellular process	*BMP1, PTBP1, SRSF11, ESR1, HNRNPA, TGFB1, IGF1R, APP, BGN, AP2A1, HNRNPUL1,HGS, LOX, ADAMTS2, EPN1, AP2M1*
6807	2.09E-02	8	Nitrogen compound metabolic process	*APP, BGN, HNRNPUL1, SRSF11, PTBP1, ESR1,TGFB1, HNRNPA*
44260	2.24E-02	10	Cellular macromolecule metabolic process	*IGF1R, APP, BGN, HNRNPUL1, SRSF11, PTBP1, ESR1, LOX, TGFB1, HNRNPA*
9653	2.24E-02	6	Anatomical structure morphogenesis	*IGF1R, APP, BGN, BMP1, ESR1, TGFB1*
44238	2.30E-02	12	Primary metabolic process	*IGF1R, APP, BGN, BMP1, HNRNPUL1, SRSF11, PTBP1, ESR1, LOX, ADAMTS2, TGFB1, HNRNPA0*
6139	2.30E-02	7	Nucleobase, nucleoside, nucleotide and nucleic acid metabolic process	*APP, HNRNPUL1, SRSF11, PTBP1, ESR1, TGFB1, HNRNPA0*
48731	2.38E-02	8	System development	*IGF1R, APP, BGN, BMP1, ESR1, LOX, ADAMTS2, TGFB1*
90304	3.05E-02	6	Nucleic acid metabolic process	*APP, HNRNPUL1, SRSF11, PTBP1, ESR1, HNRNPA0*
48856	3.18E-02	8	Anatomical structure development	*IGF1R, APP, BGN, BMP1, ESR1, LOX, ADAMTS2, TGFB1*
34641	3.25E-02	7	Cellular nitrogen compound metabolic process	*APP, HNRNPUL1, SRSF11, PTBP1, ESR1, TGFB1, HNRNPA0*
8152	3.91E-02	12	Metabolic process	*IGF1R, APP, BGN, BMP1, HNRNPUL1, SRSF11, PTBP1, ESR1, LOX, ADAMTS2, TGFB1, HNRNPA0*
7275	4.02E-02	8	Multicellular organismal development	*IGF1R, APP, BGN, BMP1, ESR1, LOX, ADAMTS2, TGFB1*

^a^x represents the number of differentially expressed genes in each GO term.

FDR, false discovery rate.

### Pathway enrichment analysis of interaction network

GENECODIS was used to perform pathway enrichment analysis of all differentially expressed genes in the interaction network, and five pathways were significantly enriched (Table 2). The pathway of hsa04510: focal adhesion, involving 21 differentially expressed genes was the most significant (FDR = 2.78E-09). The other significantly enriched pathways were the neurotrophin signaling pathway, regulation of actin cytoskeleton, pathways in cancer and the MAPK signaling pathway.

**Table 2:** List of all the differentially expressed genes in the enrichment pathway

**ID**	**Term**	**x**^**a**^	**FDR**	**Genes**
hsa04510	Focal adhesion	21	2.78E-09	*TLN1, ROCK2, ERBB2, ITGA11, ELK1, PXN, MYL9, VEGFB, CDC42, IGF1R, RAC2, PAK2, FYN, GSK3B, ILK, COL6A2, COL6A1, SHC1, COL1A1, ZYX, PARVB*
hsa04722	Neurotrophin signaling pathway	11	2.10E-04	*CDC42, RPS6KA2, MAP2K2, CAMK2G, GSK3B, SORT1, SHC1, MAPKAPK2, FOXO3, YWHAE, ARHGDIA*
hsa04810	Regulation of actin cytoskeleton	12	0.0045	*FGFR1, CDC42, ARHGEF1, PAK2, RAC2, ROCK2, MAP2K2, ITGA11, ITGB2, PXN, MYH10, MYL9*
hsa05200	Pathways in cancer	15	0.007044	*FGFR1, RXRB, MAP2K2, ERBB2, SMAD3, BCL2L1, DAPK3, TGFB1, VEGFB, IGF1R, CDC42, RAC2, GSK3B, RARA, TRAF4*
hsa04010	MAPK signaling pathway	12	0.021024	*FGFR1, CDC42, PAK2, RAC2, RPS6KA2, MAP2K2, MAP3K2, JUND, ELK1, MAPKAPK2, PPP3CA, TGFB1*

^a^x represents the number of differentially expressed genes in each pathway.

FDR, false discovery rate.

## Discussion

By the comparison of gene chips from five osteoporosis patients and four normal samples of bone marrow stem cell, we identified genes (*IL17RC*, *COL1A1*, and *ESR1*) that directly interact with the *OPG* gene.

The *IL17RC* (the interleukin 17 receptor C) gene is a growth factor that encodes a single-pass type I membrane protein as extracellular antagonists to cytokine signaling [15]. IL-17s and their receptors (IL17RC) produced in response to compressive force may affect osteoclastogenesis through the expression of RANKL and OPG [16]. IL-17RC can also promote bone and joint damage through induction of matrix metalloproteinases and osteoclasts, and stimulate osteoclastic resorption through osteoblasts by inducing receptor activator of nuclear factor κB ligand (RANKL) expression [17]. The mechanism by which *IL17RC* interacts with *OPG* needs further study.

COL1A1 (collagen Type I) is a constituent of the extra cellular matrix in connective tissue of bone, skin, tendon, ligament and dentine. It is mostly produced and secreted by osteoblasts and fibroblasts. Mutations in this gene are associated with osteogenesis imperfecta types I to IV, idiopathic osteoporosis and Caffey Disease [18]. In a population-based sample of 1,778 postmenopausal women, COLIA1 genotypes of G/G homozygotes (SS), G/T homozygotes (Ss), and T/T homozygotes (ss) is associated with reduced bone density and predisposes women to osteoporotic fractures [19]. COLIA1 Sp1(G-->T) polymorphism appears to be an important marker for low bone mass and vertebral fracture, raising the possibility that genotyping at this site may be of value in identifying women who are at risk of osteoporosis [20].

ESR1 (estrogen receptor 1) localizes to the nucleus and plays a role in tissues such as bone, and is involved in pathological processes including osteoporosis, endometrial cancer, and breast cancer. The genes (*ESR1*, *BMP1*, and *IRS1*), which are differentially expressed in the tibiae of wild type (WT) mice have recognized roles in bone metabolism or have been linked previously to osteogenesis [21]. Functional annotation showed differential expression of *ESR1*, *ESR2*, *PGR* and *BGN* genes related to estrogen metabolism and organ development and that these four genes can interact with each other; this interaction was confirmed by immunohistochemistry [22]. ESR1 can regulate bone metabolism through genome-wide association studies (GWAS) and it inhibits osteoporosis as an estrogen receptor [23]. Bioinformatics analysis revealed that a number of differentially expressed genes, including *ESR1* gene, are predicted to target genes known to be important in mammalian gonadal development [24]. Polymorphisms at *COL1A1* and *TGFB1* and haplotypes at *COL1A1* and *ESR1* were found to be associated with bone mineral density (BMD) in a cohort of postmenopausal Spanish women. Moreover, *COL1A1* polymorphisms showed significant interactions among them and with the *VDR* 3′ polymorphisms [25].

The expression of these three genes in bone marrow mesenchymal cell is expected to be used in developing biomarkers for detecting osteoporosis and for screening osteoporosis risk groups. Meanwhile, the *IL17RC*, *COL1A1*, and *ESR1* genes are related to bone protection, and the OPG gene is identified as balancing bone metabolism by osteoclast modification. In conclusion, our results indicate that the *IL17RC*, *COL1A1*, and *ESR1* genes can regulate bone metabolism by the protection of the *OPG* gene in bone marrow mesenchymal cells.

The hsa04510: focal adhesion was the most significant enriched pathway in the interaction network. Focal adhesions play a critical role in cell survival, migration and in sensing physical force. The focal adhesion pathway controls focal adhesion dynamics and can mediate reparative bone formation *in vivo* and osteoblast mechanotransduction *in vitro*[26]. Osteogenic differentiation is more prevalent in mesenchymal stem cells with a stiff, spread actin cytoskeleton and with greater numbers of focal adhesions. Both adipogenic differentiation and chondrogenic differentiation are encouraged when mesenchymal stem cells have a spherical morphology associated with a dispersed actin cytoskeleton with few focal adhesions. Different mechanical stimuli can be implemented to alter these cytoskeletal patterns and to encourage mesenchymal stem cell differentiation to the desired lineage [27].

The bone metabolism of normal adults is in a dynamic equilibrium; osteoblasts synthesize new bone and osteoclasts resorb old bone. If this balance is broken *in vivo*, osteoporosis is caused by insufficient bone formation and/or bone over-resorption [28]. Research shows that the main pathogenesis of osteoporosis is due to abnormal osteoblast activation and proliferation, and when bone absorption is more than bone formation, this negative bone metabolism leads to osteoporosis [29]. However, the combination of RANK and RANKL can be blocked by OPG, because OPG could competitive bind with RANKL and tumor necrosis factor-related apoptosis inducing ligand (TRAIL), thus osteoclast differentiation and maturation were inhibited, and osteoclast apoptosis was induced. Therefore, OPG plays a key role against osteoporosis [30]. Osteoprotegerin, a soluble member of the superfamily of tumor necrosis factor receptors, is normally secreted into marrow spaces by cells derived from mesenchyme. Osteoprotegerin acts as a decoy for osteoclast differentiation factor, which is ‘both necessary and sufficient for osteoclast development’ and is a critical regulator of postnatal skeletal development and homeostasis in humans [5].

The three main mechanisms of osteoporosis are an inadequate peak bone mass, excessive bone resorption and inadequate formation of new bone during remodeling [31]. Therefore, to increase osteoblastic cells we can improve supplementation of osteoblastic cells, or induce stem cells to differentiate into osteoblastic cells for the treatment of osteoporosis.

## Conclusions

By the comparison of gene chips from five osteoporosis patients and four normal samples of bone marrow stem cell, we identified genes (*IL17RC*, *COL1A1*, and *ESR1*) that directly interact with the *OPG* gene. Functional and pathway enrichment analyses revealed that organ development and focal adhesion were significantly dysregulated in osteoporosis patients. The expressions of *IL17RC*, *COL1A1*, and *ESR1* in bone marrow mesenchymal cell are expected to be used in developing biomarkers for detecting osteoporosis and for screening osteoporosis risk groups. However, further studies are still needed to confirm our results because our study is based on microarray generated from small sample size.

## Abbreviations

FC: Fold change; FDR: False discovery rate.

## Competing interests

The authors declare that they have no competing interests.

## Authors’ contributions

XMW ,SZG YLY and EPL carried out the molecular genetic studies. JL and WG Analyzed the data. XM GHS, CY and KNX participated in the sequence alignment and drafted the manuscript. All authors read and approved the final manuscript.

## Authors’ information

Xiaoming Wu, Shuzhang Guo and Guanghao Shen are co-first authors.
